# Conductive Hairy Particles With Homogeneous and Janus Design as Carrier Materials for the Efficient Immobilization of Unspecific Peroxygenases

**DOI:** 10.1002/biot.70078

**Published:** 2025-07-16

**Authors:** Alexander Karich, Hongtao Cai, Anne Linhardt, Anila Antony, Christiane Liers, René Ullrich, Fabian Schwaderer, Johannes Kalmbach, Katrin Scheibner, Martin Hofrichter, Alla Synytska

**Affiliations:** ^1^ Technische Universität Dresden IHI Zittau Zittau Germany; ^2^ Functional Polymer Interfaces Group Bayerisches Polymerinstitut (BPI) Universität Bayreuth Bayreuth Germany; ^3^ Brandenburgische Technische Universität Cottbus‐Senftenberg Senftenberg Germany

**Keywords:** Ag particles, core shell particles, enzyme immobilization, Janus particles, PDMAEMA, polymer brushes, unspecific peroxygenases

## Abstract

Efficient immobilization of unspecific peroxygenases (UPOs) on hairy particles with homogeneous and Janus design possessing conductive core and polymeric shell was demonstrated. PDMAEMA brushes (hairs) with controlled chain lengths were successfully grown from the conductive silver particles allowing further immobilization of enzymes and keeping their activity. The Janus design of the synthesized particles maintained the conductivity of the core material. Enzyme immobilization on brush‐modified particles was first carried out with two laccases as model enzymes [*Trametes versicolor* (*Tve*Lac) and *Pycnoporus cinnabarinus* (*Pci*Lac)] and then successfully extended to UPOs [two wild‐type and one recombinant UPO from *Marasmius rotula* (*Mro*UPO) and *Agrocybe aegerita* (*Aae*UPO, r*Aae*UPO)]. The most efficient immobilization was achieved for *Mro*UPO on Janus particles. The enzyme loading is reversible and could be successfully repeated after cleaning of the particles. Thus, the successful immobilization of an *Mro*UPO on conductive hairy Janus particles was demonstrated for the first time. The major advantage of the proposed approach lies in the reusability of the enzyme and its carrier, as well as in the use of conductive core materials, which could be promising, for example, as material for electrochemical biosensors in future.

## Graphical Abstract and Lay Summary

Efficient immobilization of unspecific oxidoreductases on hairy core‐shell particles with homogeneous and Janus design possessing conductive core material and polymeric brush shell is demonstrated. PDMAEMA brushes with controlled chain lengths (4, 11, and 23 nm) are successfully grown from conductive silver particles. Hairy particles with Janus design maintained the conductivity of the hybrid carrier material.

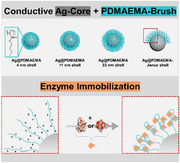

## 1 Introduction

Due to their high specificity and sensitivity, enzymes such as unspecific peroxygenases (UPOs; EC1.11.2.1) have great potential for environmental monitoring and especially for the use in electrochemical biosensors [1]. However, it is challenging to immobilize them on electrodes in sufficient quantities to maintain or even improve their activity, long‐term stability, and robustness under different reaction conditions [2]. Therefore, the immobilization of such enzymes on a carrier with high protein loading capacity and protective properties is of great biotechnological interest. UPOs have already been immobilized using various methods ranging from covalent and ionic immobilization to entrapment in gels and CLEA [3]. Immobilization systems based on covalent binding of a recombinant UPO of *Agrocybe aegerita* (r*Aae*UPO) to different carriers (e.g., polyacrylate; aminomethacrylate) led to immobilization yields of 55% and 73%, respectively, but with low active enzyme yield of merely 2% [4] UPO immobilization in a microfluidic concept was proven by Gkantzou et al. [5] with an activity recovery of 51% and combined with high TTN number. The electrostatic immobilization of UPOs of *Agrocybe aegerita* and *Marasmius rotula* (*Aae*UPO and *Mro*UPO, respectively) to a matrix of gold nanoparticles embedded in chitosan on the surface of a glassy carbon electrode was also successfully performed and could be used for the detection of UPO‐specific substrates in the micromolar range [6]. So far, there is little knowledge about the immobilization of UPOs on hybrid core‐shell particles with hairy or brush‐like polymer shell, although these particles are promising candidates due to their customizability and the already known high loading capacity.

One successful immobilization strategy uses the immobilization of proteins in polymer brushes by electrostatic interactions. In contrast to other immobilization strategies, polymer brushes offer the benefit of high protein loading that preserves enzyme activity due to the hydrated 3D environment within the brush structure [7]. Several studies have shown that polyelectrolyte brushes are a useful surface modification for the immobilization of proteins and enzymes by preserving the structure and biological activity [8]. Although brushes have clear advantages over other immobilization strategies, their practical application is limited due to difficulties in immobilizing them on electrodes [9]. A better option is the immobilization of enzymes in polymer brushes on particle‐based systems [8, 10]. The particles have a large surface area in relation to their volume, which means that a higher concentration of enzymes can be immobilized. This increased enzyme loading leads to increased signal generation in response to the target analyte, which ultimately increases the sensitivity of the biosensor [11]. Moreover, for later application in electrochemical biosensors, these carriers should combine conductivity and high enzyme loading to enhance electron transfer. Polymers and inorganic particles can be combined through surface modification methods like grafting from or grafting to [12]. By adapting the polymer and the core material, the properties of the resulting particle systems, such as adhesion, hydrophilicity/hydrophobicity, enzyme loading capacity and electron transfer, can be controlled regarding the desired application. Consequently, a variety of functions can be achieved on hybrid particles through targeted polymerization, polymer modification, and the choice of a suitable core material.

Immobilization of enzymes on particle systems is of general interest particularly in biotechnological, chemical/industrial, and environmental applications [10e]. The key benefits are the reusability of the enzymes, increased stability, controlled reaction conditions, and enhanced catalytic efficiency. Especially, the immobilization of UPOs is of great interest for applications as biocatalyst or as detecting elements in electrochemical biosensors relevant for environmental analysis and drug monitoring (e.g., detection of pharmaceutical pollutants).

**FIGURE 1:**
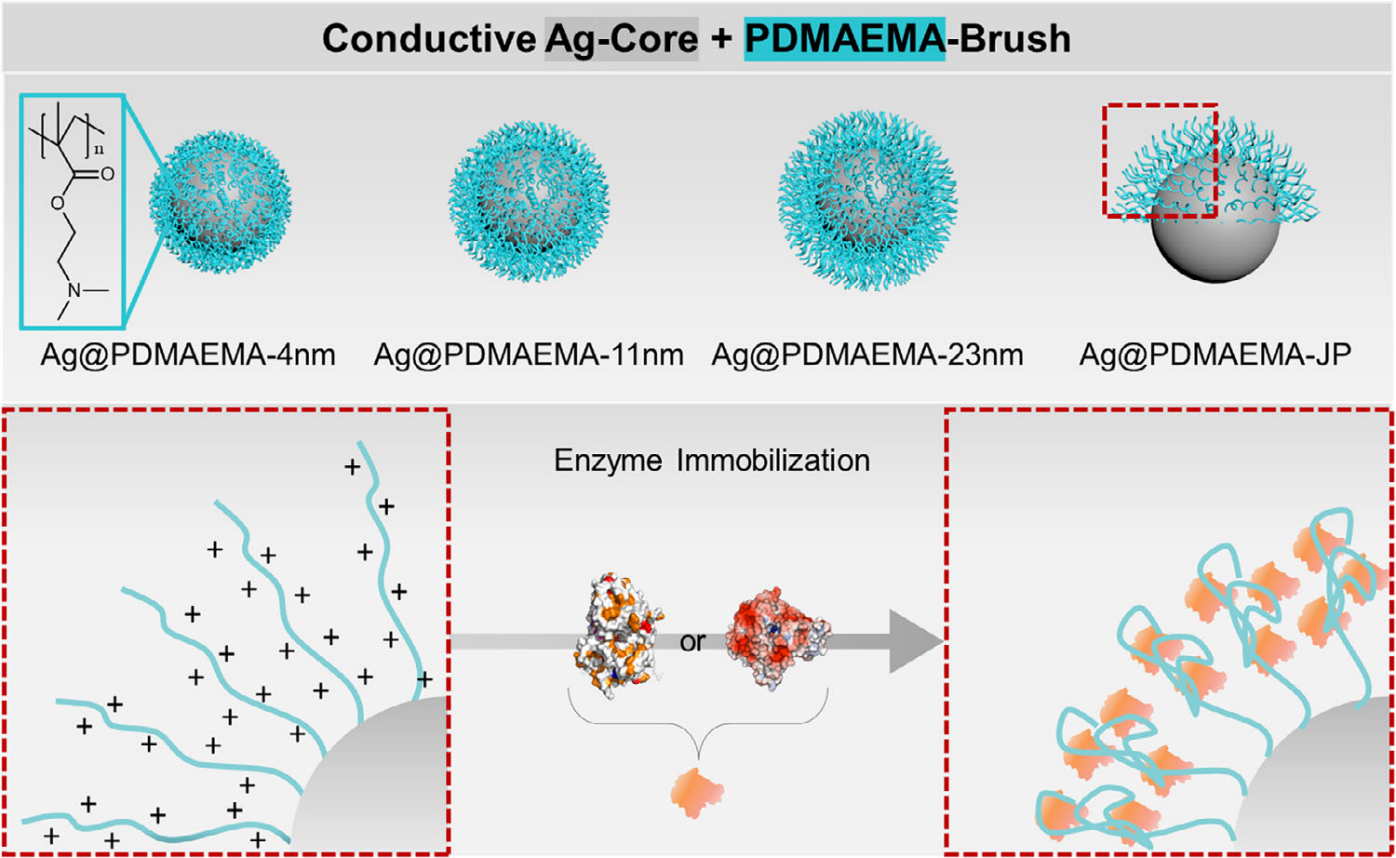
Scheme illustrating the studied particle libraries with homogeneous and Janus design and enzyme immobilization by electrostatic interactions.

Herein the immobilization of two fungal laccases (EC 1.10.3.2; *Trametes versicolor*—*Tve*Lac and *Pycnoporus cinnabarinus*—*Pci*Lac) and three UPOs (two wild‐type proteins—*Aae*UPO, *Mro*UPO and a recombinant protein—r*Aae*UPO) in polyelectrolyte brushes on silver (Ag) particles, is described for the first time (Figure 1). Therefore, two core‐shell Ag‐based systems are investigated: One fully covered with PDMAEMA brushes of different chain lengths and the other a partially covered Ag‐PDMAEMA‐Janus particles (Ag@PDMAEMA‐JP).

## 2 Results and Discussion

Four different Ag‐based particle systems with PDMAEMA brushes were synthesized and investigated for the immobilization of laccase (EC 1.10.3.2) and UPOs (EC 1.11.2.1). The thickness of the brush‐like particle shell was varied by the amount of monomer added for polymerization. To keep the core material accessible, a Janus‐shaped particle (JP) partially covered with a polymer brush was also synthesized from a wax‐water Pickering emulsion using the well‐known colloidosome technique [8, 13]. This is of great interest with regard to one of the planned applications of these particles as electrode material for electrochemical biosensors.

**FIGURE 2:**
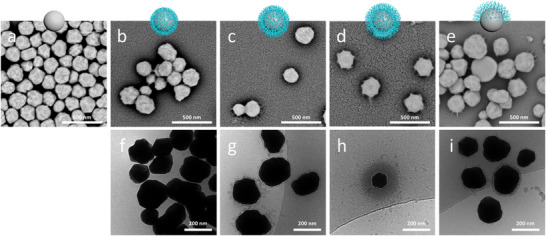
Representative SEM images of (a) native Ag particles, (b) Ag@PDMAEMA‐4 nm, (c) Ag@PDMAEMA‐11 nm, (d) Ag@PDMAEMA‐23 nm, and (e) Ag@PDMAEMA‐JP. Representative TEM images of (f) Ag@PDMAEMA‐4 nm, (g) Ag@PDMAEMA‐11 nm, (h) Ag@PDMAEMA‐23 nm, and (i) Ag@PDMAEMA‐JP.

All particles were characterized by scanning (SEM) and transmission electron microscopy (TEM), dynamic light scattering (DLS), zeta potential and thermogravimetric (TGA) measurements. The TGA also allowed the calculation of the amount of polymer grafted onto the particle surface and thus the thickness of the polymer shell (in the dry state). The calculation was carried out for spherical particles, as previously published for silicon dioxide particles [14], since the obtained Ag particles are very close to this spherical system. All results are summarized in Table 1. Electron microscopy is shown in Figure 2, showing the increase in polymer shell thickness as well as the Janus‐character of the Ag@PDMAEMA‐JP particle. The increase in hydrodynamic volume and polymer fraction is further proved by DLS and TGA. Furthermore, the isoelectric point (IEP) of the particle changed from pH 3.0 to values around 9.0 (Figure 5), indicating successful polymer grafting due to the polycationic character of PDMAEMA.

**TABLE 1:** Summary of investigated particle systems.

Sample ID	Dh [nm]^DLS^ (*n* = 3)	Shell thickness [nm]^TGA^	Mn [g/mol]^GPC^ (*n* = 2)	ζ‐potential at pH 4 [mV]	ζ‐potential at pH 6 [mV]	IEP, pH
Ag	190 ± 6	—	—	−11	−30	3,0
Ag@PDMAEMA‐4nm	296 ± 19	4	—	39	39	9.0
Ag@PDMAEMA‐11nm	479 ± 11	11	—	46	42	9.3
Ag@PDMAEMA‐23nm	810 ± 23	23	79,050 ± 450	49	41	9.4
Ag@PDMAEMA‐JP	736 ± 32	8^a^	30,800 ± 0	36	34	9.2

^a^ Calculated for a total polymer covered particle.

### 2.1 Immobilization of Laccase on the Hybrid Particle Systems

**FIGURE 3:**
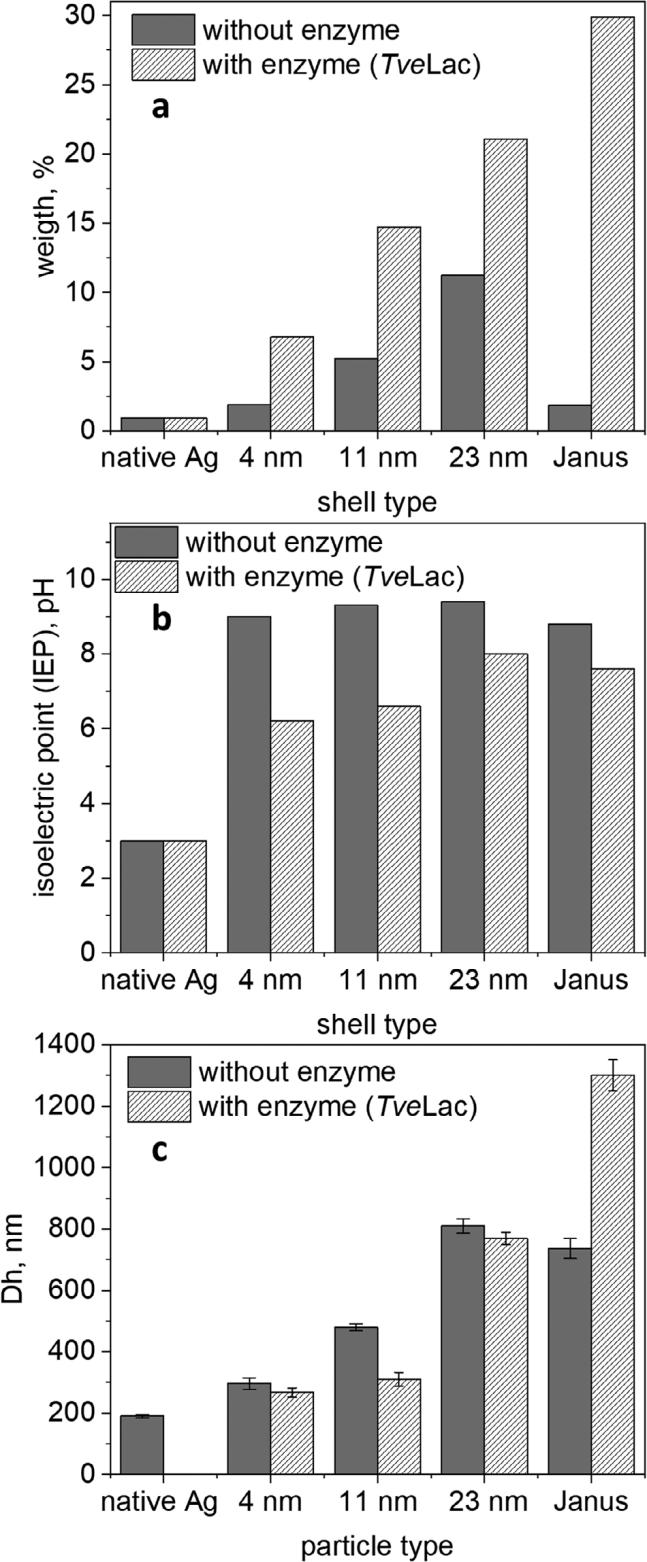
Mass determined by TGA (a), isoelectric point determined by electrokinetic studies (b), hydrodynamic diameter (D_h_) determined by DLS measurement (c, *n* = 3) before and after enzyme loading with Laccase *Tve*Lac.

**FIGURE 4:**
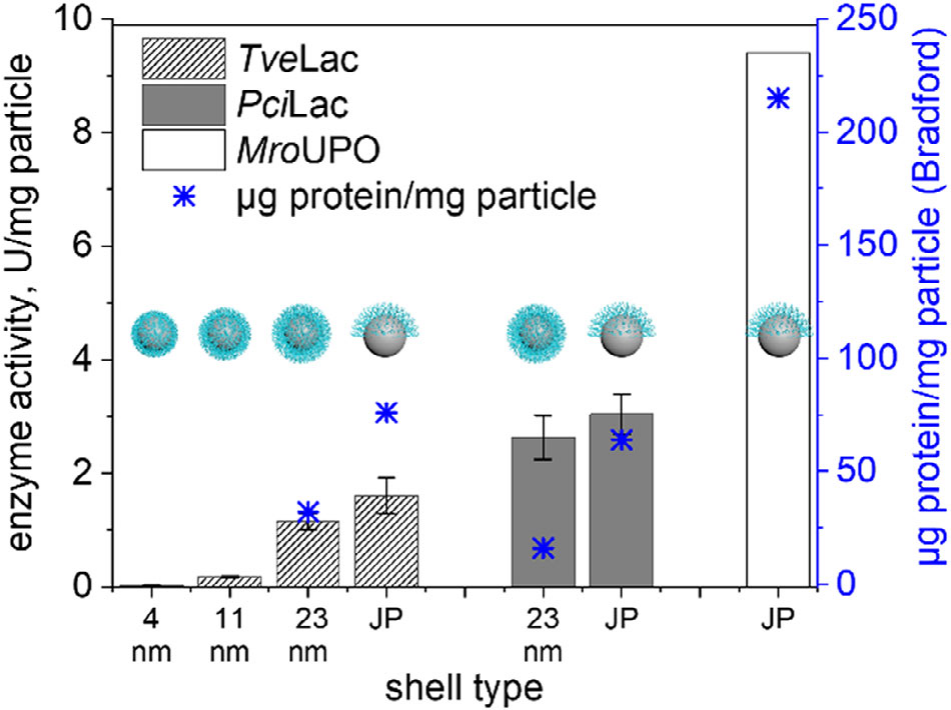
Enzyme activities of immobilized *Tve*Lac (U/mg particle) for all investigated Ag@PDMAEMA particle types and of *Pci*Lac on Ag@PDMAEMA‐23 nm and Ag@PDMAEMA‐JP detected with ABTS as substrate as well as *Mro*UPO activity on Ag@PDMAEMA‐JP detected with veratryl alcohol (VA). The mass of protein immobilized per particle was determined by the Bradford assay. Immobilization of laccase and *Mro*UPO was performed at pH 4.0 and 6.0, respectively.

The laccase preparations of *T. versicolor* (*Tve*Lac) and *P. cinnabarinus* (*Pci*Lac) were used as model proteins to study the efficiency of immobilization on the new JP systems and to compare them with previous work, in which *Tve*Lac was successfully immobilized on similar particles with a non‐conductive silica core [10b,c]. Figure 3 shows the changes in mass, isoelectric point (IEP) and hydrodynamic diameter before and after the immobilization of *Tve*Lac. Since successful immobilization increases the combustible portion of the particle system, the mass loss, determined by TGA analysis, increased (Figure 3a). As the immobilized enzyme covered the polymer batch, the IEP changed, as shown in Figure 3b. The decrease in hydrodynamic diameter can be explained by the collapse of the polymer brush during enzyme immobilization (Figure 1) and by the lower swelling properties. The loading capacity for enzymes increases with the length of the polymer chains and thus with the thickness of the polymer shell (Figure 4a). The enzyme activity and loading capacity of the Janus particles was significantly higher than that of the fully polymer‐coated particles. This can be attributed to the fact that the polymer chains expand into the area where no polymer is grafted, hence reducing steric barriers. Immobilization on bare Ag particles was tested as a reference sample for enzyme immobilization without success. It is therefore not assumed that the enzyme was immobilized on the Ag@PDMAEMA‐JP surfaces without a polymer brush.

Particles with low shell thickness (4 and 11 nm) showed the lowest enzyme activity (Figure 4). It is assumed that the relatively small polymer fraction available for the enzymes and the poor dispersibility of these particles are responsible for the low enzyme loading efficiency, since these particles have a higher tendency to aggregate and sediment. In terms of dispersibility and enzyme loading capacity, Ag@PDMAEMA‐23 nm and Ag@PDMAEMA‐JP turned out to be the most suitable particles for immobilization studies. Therefore, these particles were used for further investigations and the immobilization of UPOs.

### 2.2 Immobilization of UPOs on the Hairy Hybrid Particles at Different pH

The immobilization of UPOs in the polymer brush of Ag@PDMAEMA modified particles was studied for three different UPO types (Table 2; *Aae*UPO, r*Aae*UPO, and *Mro*UPO). The immobilization of all three UPOs on the Ag@PDMAEMA polymer brushes led to insufficient results at pH 4.0, and almost no enzymatic activity could be detected with these particles (Table 2). Only the *Mro*UPO was successfully immobilized on two particle systems (Ag@PDMAEMA‐23 nm and Ag@PDMAEMA‐JP) and only at a pH of 6.0. Although immobilization on Ag@PDMAEMA‐23 nm led to reproducible results with moderate enzyme loads (4.9 U_VA_/mg particle), the final preparation showed poor dispersibility and a tendency to aggregate and sediment. These particle systems were excluded from further investigations. However, promising immobilization was achieved for the loading of Ag@PDMAEMA‐JP with *Mro*UPO at pH 6.0, as reflected by high enzyme activities (9.7 U_VA_/mg particle) and good particle dispersion (Table 2). These results were reproducible in further immobilization batches and led to an average UPO activity of 9.4 U_VA_/mg particle (Table 2).

**TABLE 2:** Enzymatic activity of three UPOs after immobilization on three different Ag@PDMAEMA systems.

		Enzymatic activity [U_VA_/mg particle] detected with VA assay		
Enzyme	Dh [nm]^DLS^	Ag@PDMAEMA‐4nm	Ag@PDMAEMA‐23nm	Ag@PDMAEMA‐JP
*Aae*UPO	pH 4.0	0.0^a^	0.4^a^	0.13^a^
	pH 6.0	n.d.	n.d.	0.04^a^
r*Aae*UPO	pH 4.0	0.0^a^	0.0	0.0
	pH 6.0	0.0^a^	0.0	0.0
*Mro*UPO	pH 4.0	0.0^a^	0.0	0.6
	pH 6.0	0.1	4.9	9.7

Abbreviation: n.d., no data available.

^a^ Moderate to strong coagulation of particles after immobilization.

Due to the challenging physicochemical conditions of particle preparation, there is a systematic error of up to 30%, especially in enzyme particle systems with strong coagulation. This is mainly due to lengthy and heterogeneous treatment of the particles and the resulting handling errors. Specific errors result from the following practices: (i) Portioning of the particles from the initial batch into smaller aliquots, for example, when preparing dilutions for activity measurement and protein determination. (ii) The in some cases strong coagulation of particle systems or particle/enzyme/pH combinations and the adsorption of particles on working utensils (tubes and pipette tips) may lead to further handling errors. Therefore, the measurement of enzyme activity was somewhat biased in such cases, since it was not possible to take homogeneous aliquots of the sample and therefore the settled particles had to be used for the measurement. As a result, either no activity could be measured, or the activity was overrepresented. This was especially the case with the measurements of the *Aae*UPO samples, where there was always coagulation and thus sedimentation (Table 2).

### 2.3 Comparison of Laccase and UPO Immobilization

**FIGURE 5:**
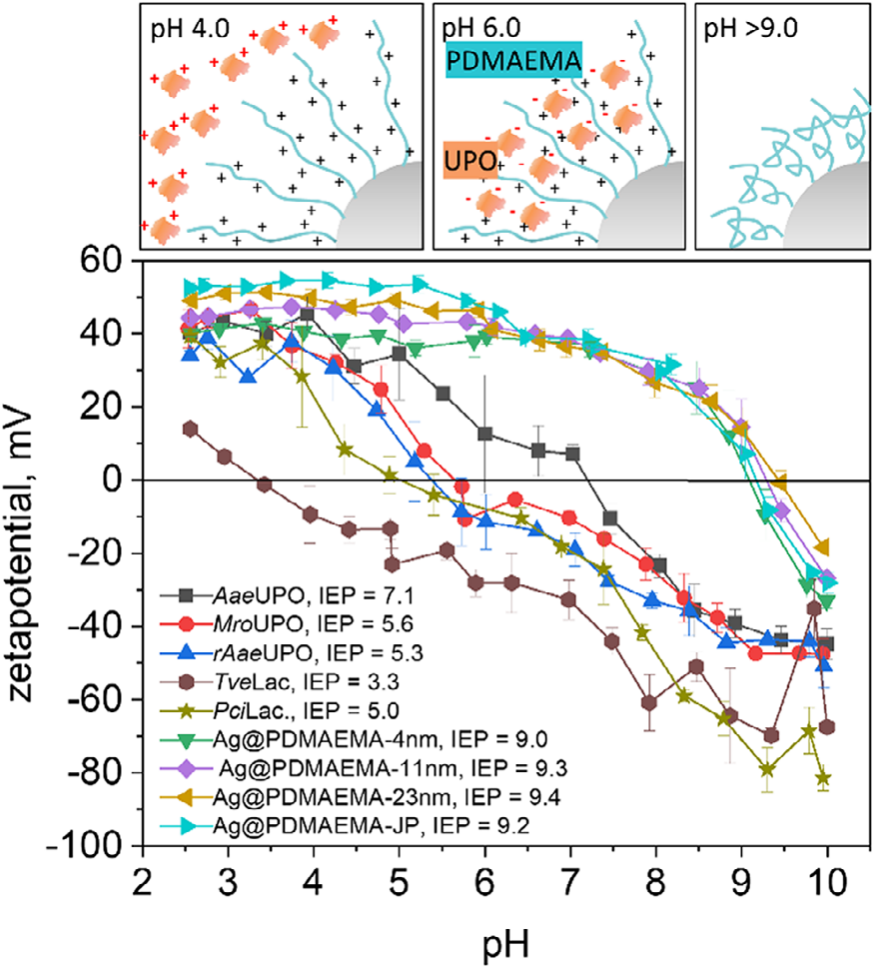
Schematic representation of the hybrid particles at different pH and zeta potential of the synthesized particles systems and the studied enzymes (*Tve*Lac, *Pci*Lac, r*Aae*UPO, *Aae*UPO, and *Mro*UPO) in dependence of pH. In aqueous solutions, at pH below 9.0, the polymer chains are positively charged and show a brush‐like geometry due to electrostatic repulsion. At higher pH, (above pH 9.0), brushes tend to collapse and the PDMAEMA chains form coils. For proper immobilization, a pH should be chosen at which the polymeric particle chains are positively charged, and the enzyme surface is negatively charged.

The immobilization studies with both *Tve*Lac and the UPOs (*Aae*UPO, r*Aae*UPO, *Mro*UPO) clearly showed a significant influence of the pH on the immobilization yield. While the immobilization of *Tve*Lac was most successful at pH 4.0, the immobilization of the UPOs at this pH did not result in detectable UPO activity on the particles; only at pH 6.0 they could be successfully immobilized. This pH dependence can be explained by the different isoelectric points (IEPs) of enzyme proteins. The IEP of the PDMAEMA particles is around 9.0, which means that the particles are positively charged over a wide pH range (<pH 9.0) and thus offer the possibility of binding and immobilizing negatively charged enzyme proteins by electrostatic interactions [10b,c]. Figure 5 shows the zeta potential of all enzymes analyzed and the respective hybrid particle systems as a function of pH. The enzyme proteins studied are negatively charged within different pH ranges. The corresponding IEPs of the investigated enzymes are 3.6, 5.0, 5.3, 5.6, and 7.1 for *Tve*Lac, *Pci*Lac, r*Aae*UPO, *Mro*UPO, and *Aae*UPO, respectively. This partly explains the different behavior of these enzymes during immobilization at different pH. At pH 4.0, both the polymer brushes and the UPOs would be positively charged. Consequently, electrostatic repulsion hinders protein immobilization. The high IEP of *Aae*UPO at pH 7.1 resulted in very low immobilization of this enzyme at pH 4.0 and 6.0, as it was still positively charged at these pH values. The IEPs of *Mro*UPO and r*Aae*UPO around pH 5.0 also explain why immobilization was less successful at pH 4.0 than at pH 6.0, as the proteins had a positive surface charge at pH below 5.0 and a negative surface charge above 5.0 (compare Table 2 and Figure 5). In contrast, the immobilization of *Tve*Lac at pH 4.0 was more successful, as this enzyme already has a negative surface charge at pH values above 3.3.

Contradictory results were obtained for the immobilization of *Pci*Lac on Ag@PDMAEMA‐23 nm and Ag@PDMAEMA‐JP (Figure 4 and Table 3). Although the IEP of *Pci*Lac is around pH 5.0, a significantly higher loading with active enzyme was found for this preparation on the polymer brushes than for *Tve*Lac. It is possible that the differences in the immobilization success were also due to the purity and origin of the respective enzyme preparations (e.g., regarding the degree of glycosylation). While *Tve*Lac is a commercial recombinant protein (actually r*Tve*Lac) with a rather low specific activity (0.33 U_ABTS_/mg), *Pci*Lac is a highly pure wild‐type enzyme (295 U_ABTS_/mg) that was homologously produced with the native fungal species. The fact that *Pci*Lac was used with a degree of purification that was three orders of magnitude higher led to a better enzyme/particle load compared to *Tve*Lac. This is also reflected by the specific enzyme activities of the two final particle preparations, namely 35.9 U_ABTS_/mg compared to 164.4 U_ABTS_/mg for *Tve*Lac and *Pci*Lac, respectively. An interesting side effect becomes apparent when the specific enzyme activities are compared before and after immobilization. The value for *Tve*Lac increased by a hundred‐fold, while that for *Pci*Lac decreased by about 50%. This means that active *Tve*Lac molecules tend to bind more specifically to the particles than protein impurities of the enzyme preparation at a pH of 4.0. In contrast, the highly pure *Pci*Lac did not bind more strongly to the particles and, in fact, lost specific activity, possibly due to steric interference between the enzyme molecules and the particles (e.g., by interacting with the copper‐containing enzyme's active site).

**TABLE 3:** Protein load on hybrid particles according to Bradford and the resulting specific activities on the particles.

Enzyme	Particle	Batch	µg protein/mg particle	Enzyme activity [U/mg particle]^b^	Specific activity on particles [U/mg protein]^b^
*Tve*Lac	Ag@PDMAEMA‐23nm	2	32	1.15	35.9
*Tve*Lac	Ag@PDMAEMA‐JP	2	76	1.16	15.3
*Tve*Lac	Ag@PDMAEMA‐JP	3	66	0.97	14.7
*Pci*Lac	Ag@PDMAEMA‐23nm	2	16	2.63	164.4
*Pci*Lac	Ag@PDMAEMA‐JP	3	64	3.03	47.3
*Mro*UPO	Ag@PDMAEMA‐JP	2	215	9.70	45.17
*Mro*UPO^a^	Ag@PDMAEMA‐JP	2	167	7.00	41.9

^a^ Mean value of the reused particles.

^b^ Laccase activity was detected with ABTS assay, UPO activity with VA assay.

It is noteworthy that the protein load on hairy Ag@PDMAEMA‐JP Janus particles with *Mro*UPO is about three times higher than that with laccase. However, these data must be regarded with caution, since laccases and UPOs were immobilized at different pH values, that is, pH 4.0 and pH 6.0, respectively. The large difference in protein loads per particle may also be related to the protein structure and shape of the corresponding enzymes. *Mro*UPO is known to form dimers or even tetramers under physiological conditions [15]. This could be a fact that positively influences immobilization. Different glycosylation is certainly another factor that influences protein loading on the particles, as high glycosylation may shield the charges on the protein surface from the particle brushes [16]. Both homologously and heterologously expressed proteins can have highly variable glycosylation coats surrounding the apoprotein, but the actual extent of glycosylation for each enzyme preparation is rather difficult and laborious to determine [17].

### 2.4 Evaluation of *Mro*UPO on Hairy Ag@PDMAEMA‐JP Janus Particles

We determined 9.4 U_VA_/mg particles; (measured at pH 6) and 2.3 U_ABTS_/mg particles (measured at pH 4.5). This results in a ratio of 4.1:1 (VA:ABTS). The ratio for the pure *Mro*UPO preparation is 4.4:1 (607 U_VA_/mL: 139 U_ABTS_/mL). Hence, we conclude that the VA/ABTS ratio is not or only slightly shifted after immobilization.

Based on the activity of the particle preparation and the specific activity of the original enzyme sample (54 U_VA_/mg protein), a theoretical value of 0.174 mg of enzyme bound to 1 mg of particle was calculated. The “actual” protein loading of the best immobilization product (Ag@PDMAEMA‐JP, *Mro*UPO, pH 6.0) was determined using the Bradford assay. The average Bradford value of the immobilized UPO preparation was 2.15 mg protein/mL for 10 mg particles (after subtraction of a respective blank) which gives a final value of 0.215 mg protein/mg particle and a specific activity of 44 U_VA_/mg protein. The decrease in specific activity of the immobilized preparation by about 17% compared to the initial preparation is acceptable in terms of known activity losses during immobilization [10c]. The small loss of activity is within the expected error range for activity measurements with immobilized enzymes (up to 30%, see above), although it is not clear whether the loss was caused only by strong coagulation between the particles or also by the immobilization procedure itself. However, it is also conceivable that some of the enzyme activity is lost due to the incorrect orientation of the enzyme molecules on the polymer chains.

### 2.5 pH Dependent Reactions of *Mro*UPO Immobilized on Hairy Ag@PDMAEMA‐JP Janus Particles

**FIGURE 6:**
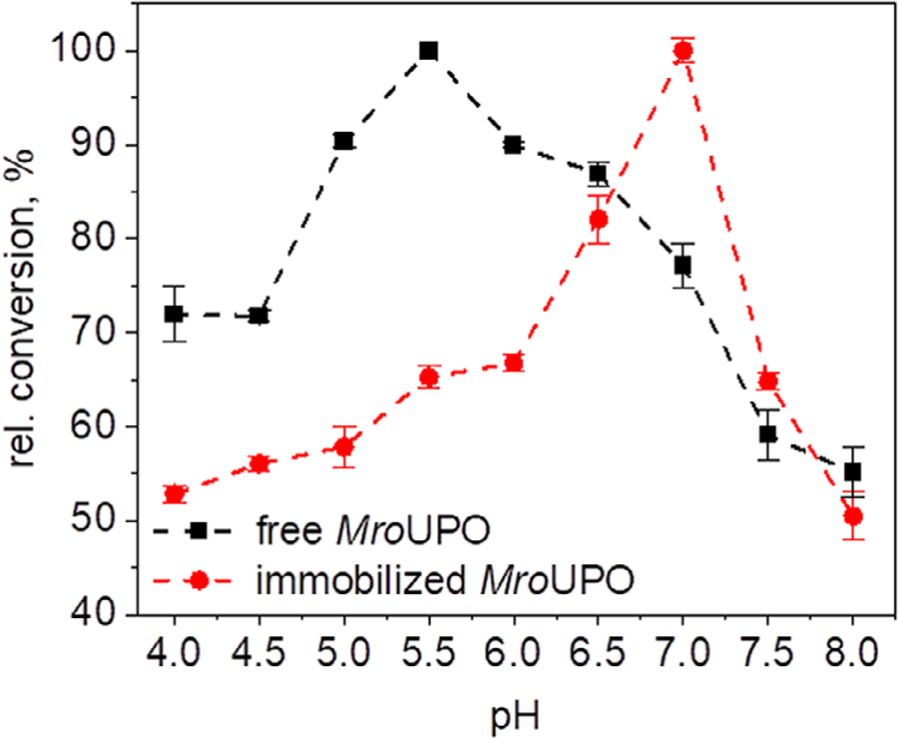
pH profiles of the relative conversion of veratryl alcohol to veratraldehyde by free *Mro*UPO (black squares) and *Mro*UPO immobilized on hairy Janus Ag@PDMAEMA‐JP (red circles).

**FIGURE 7:**
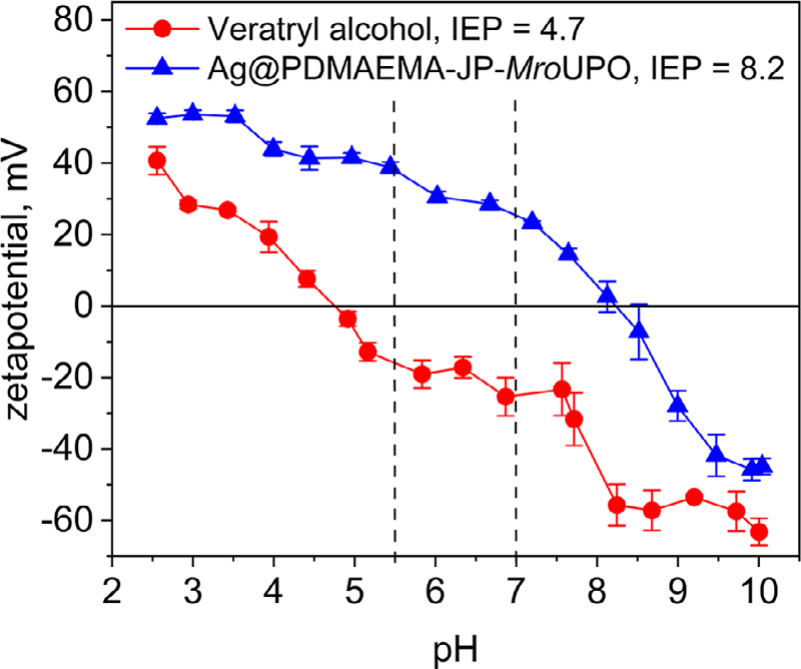
Zeta potential of veratryl alcohol and Ag@PDMAEMA‐JP with immobilized *Mro*UPO at different pH values.

The immobilization of *Mro*UPO on Ag@PDMAEMA‐JP had a significant influence on the pH profiles of *Mro*UPO‐based oxygenation reactions. The optimum shifted from pH 5.5 to pH 7.0 (Figure 6), both for oxygenation (VA oxidation) and for one‐electron oxidation (ABTS oxidation). The pH optimum of the free enzyme was 5.5 for the conversion of veratryl alcohol into veratraldehyde in aqueous solution. At lower pH (4.0–4.5), the conversion still reached approx. 75%, and at pH between 5.0 and 6.5, about 90%. At pH above 7.0, the conversion dropped markedly to below 60%. These data correspond well to those originally published by Gröbe et al. for the first purified (free) *Mro*UPO [18]. The pH optimum for immobilized *Mro*UPO, however, turned out be noticeably higher than that of the free enzyme. Thus, a VA conversion of less than 60% was achieved at pH values < 5.5. At pH 6.5, a conversion of approx. 80% was achieved and at pH 7.0, the immobilized *Mro*UPO preparation showed a distinct pH optimum. Above pH 7.5, on the other hand, the activity dropped drastically to about 65% and then to 50% at pH 8.0 (see Figure 7).

Reaction approaches with *Mro*UPO immobilized on Ag@PDMAEMA‐JP and washed in different buffers (at pH 5.5 and pH 7) prior to use did not give changed activities and no free UPO was detected in the supernatants. The shift of the pH optimum must therefore have been caused by an interaction of the particles with the enzyme and not by a pH effect that would lead to the detachment of the enzyme molecules from the particles.

Changes in the physicochemical properties such as pH profiles of immobilized fungal enzymes compared to free proteins have already been reported by several authors [19]. Keerti et al. suggested that this is due to secondary interactions between the enzyme and the polymeric matrix of the immobilizing support system [19b]. Similarly, we think that a shift in pH profile could be due to the fact that the charged surfaces of the polymer brushes and enzyme create a charged nanosphere that affects the nature of the active enzyme (e.g., by conformational change) and thus alters the pH in the immediate nano‐environment of the bound enzyme [20].

Another possible explanation could be electrostatic repulsion of the substrate and the enzyme carrier. But we exclude this for the following reasons: Electrokinetic measurements show a clear pH dependence of the surface charge of both the substrate and the enzyme carrier. Below pH 4.7, both the particle system and veratryl alcohol (VA) have a positive surface charge, and above pH 8.2, both the substrates tested, and the particle shell have a negative surface charge. It is therefore plausible that electrostatic repulsion prevents diffusion of the substrate through the polymer brush to the enzyme's active site at these pH values. However, the zeta potential measurements do not provide an explanation for the pH shift of the pH profiles for veratryl alcohol conversion, with maxima at pH 5.5 and pH 7, since VA is negatively charged at pH 4.7, whereas the Ag@PDMAEMA‐JP is positively charged up to pH 8.2. Consequently, there should be no electrostatic repulsion in this pH range.

The possibility of shifting the reaction profiles of enzymes toward more acidic or alkaline conditions by immobilization was already pointed out by Guisan et al. [21]. Furthermore, Guzik et al. postulated a change in the physicochemical properties of enzymes caused by immobilization as a possible method to modulate and improve the catalytic properties of oxidoreductases in order to increase their economic value [22].

### 2.6 Re‐Immobilization of UPOs on Cleaned and Reused Hairy Janus Ag@PDMAEMA‐JPs Particles and Variation of Initial Enzyme Activity

The initial activity for immobilization of *Mro*UPO on reused Ag@PDMAEMA‐JPs did not seemingly influence the immobilization efficiency (Table 4). Thus, the results for the three different initial UPO activities used (50, 100, and 150 U_VA_/mL) were similar, both in terms of immobilized active enzyme (7.1, 6.6, and 7.3 U_VA_/mg particle) and the amount of protein determined by the Bradford assay (183, 161, and 158 µg protein/mg particle). The activity of the particles (6.6–7.3 U_VA_/mg) was slightly lower than their activity after the first immobilization (9.7 U_VA_/mg), which indicates a certain reduction in loading capacity due to the salt treatment during the cleaning process and/or the presence of inactive enzyme residues on the particles from the first use. Upon examination, after the salt buffer treatment, the proportion of particles that could not be resuspended increased, indicating that some of the particles were damaged during the cleaning process or did not fully recover during the cleaning procedure, for example, due to residual protein contamination or inactivated enzymes. Crude enzyme preparations from fungal wild‐type strains usually contain mixtures of different isoenzymes (or protein isoforms) as well as a variety of protein impurities. Purification by anion‐exchange chromatography and size‐exclusion chromatography (as used here) cannot always remove all these impurities to yield homogenous isoenzyme fractions. The *Mro*UPO preparation used herein was not purified to the level of a homogenous isoenzyme. Thus, our results further indicate that the immobilization process does not discriminate between these specific isoenzyme fractions, probably because of their similar or even identical physicochemical properties (e.g., regarding IEF or molecular mass) [23].

**TABLE 4:** Resulting *Mro*UPO activity after re‐immobilization of cleaned and reused Ag@PDMAEMA‐JP with varying initial UPO activities.

Ag@PDMAEMA‐JP	Activity [U_VA_/mL] in 500 µL	mg particles in 1 mL (estimation)	∑U washing Steps 1–4	Activity [U_VA_/mg particle]	µg protein/mg particle^b^	Coagulation
*Mro*UPO pH 6	50	2	25	7.1	183	Low
*Mro*UPO pH 6	100	2	83	6.6	161	Low
*Mro*UPO pH 6	150	2	122	7.3	158	Low
*Mro*UPO pH 6^a^	300	10	212	9.7	215	Low

^a^ Non‐reused Ag@PDMAEMA‐JP.

^b^ Determined by the Bradford method.

## 3 Conclusion

In this work, the successful immobilization of UPOs on hairy core‐shell particles with homogeneous (fully covered by polymeric shell) and Janus design possessing a conductive core material and a polyelectrolyte shell brush has been achieved. To obtain a conductive support with polymer brushes for enzyme immobilization and protection, PDMAEMA brushes were successfully grown from a core of conductive Ag particles. Janus particle design allowed to retain the conductive properties of the core material. The physicochemical properties and immobilization behavior of Janus and fully covered polymeric particles with different chain lengths were investigated and compared. Enzyme immobilization on these particles was first established using two model enzymes, laccase from *Trametes versicolor* (*Tve*Lac) and *Pycnoporus cinnabarinus* (*Pci*Lac), and then applied to three unspecific peroxygenases [two wild‐type and one recombinant UPO from *Marasmius rotula* (*Mro*UPO) and *Agrocybe aegerita* (*Aae*UPO, r*Aae*UPO)]. *Mro*UPO on hairy Ag@PDMAEMA‐JP Janus particles proved to be the best combination resulting in 9.4 U_VA_/mg particle and 43.7 U_VA_/µg particle. It was demonstrated that these enzyme carriers are reusable. The enzyme loading is reversible and could be successfully repeated. Thus, successful immobilization of an UPO, a versatile and potentially industrially relevant biocatalyst, on conductive hairy Janus particles with polyelectrolyte brush was demonstrated for the first time. These findings offer promising approaches for future investigations and applications. Such particle systems are reusable, which is of great importance in terms of sustainability. In future, for example, enzyme‐loaded Janus particles could be used for different sequential enzyme reactions. The change in pH optimum of the enzyme bound to the Janus particles allows the modification of the substrate spectrum and thus the modulation of the catalytic potential of the enzyme‐Janus particle system. Due to their reusability, such hybrid core‐shell particle‐based materials could give a new impetus to UPO‐based catalysis both with respect to their use in biosensors and their application in biochemical synthesis. Especially the conductivity of the Ag Janus particle‐based system makes them promising candidates for electrochemical biosensing.

## 4 Materials and Methods

*Chemicals*: Tetraethyl orthosilicate (TEOS, Sigma, 99%), ammonia solution (NH_4_OH, Sigma, 28%–30% solution), ethanol abs. (EtOH, Sigma, 99.9%), copper(II) bromide (CuBr_2_, Sigma, 99.999%), (3‐aminopropyl)triethoxysilane (APTES, Sigma, 99%), a‐bromoisobutyryl bromide (BrIn, Sigma, 98%), propionyl bromide (Sigma, 97%), tris(2‐pyridylmethyl)amine (TPMA, Sigma, 98%), tin(II) 2‐ethylhexanoate (Sigma, 95%), ethyl a‐bromoisobutyrate (EBiB, Sigma, 98%), silver nitrate (Sigma, ≥99.0%), 2‐Mercaptoethanol (Sigma, ≥99.0%), 11‐Mercaptoundecyl 2‐Bromo‐2‐methylpropanoate(TCI, >95.0%), dopamine hydrochloride (Sigma, >98.0%), polyvinylpyrrolidone (PVP, Sigma, M_w_ 40000), hydrogen tetrachloroaurate(III) trihydrate (HAuCl_4_·3H_2_O, Acros, 99%), L‐ascorbic acid (Sigma, 99%), acetonitrile (Sigma, HPLC grade ≥99.9%), sodium borohydride (NaBH_4_, Sigma, 99%), dichloromethane (Acros, 99.99%), sodium acetate (Sigma ≥99.0%), and 2,2′‐azino‐bis(3‐ethylbenzothiazoline‐6‐sulfonic acid) (ABTS, Boehringer Mannheim GmbH), 3,4‐Dimethoxybenzyl alcohol (veratryl alcohol, Sigma, 96%) were used as received. 2‐(Dimethylamino)ethylmethacrylate (DMAEMA, Sigma, 98%) was passed through basic, neutral, and acidic aluminum oxide columns for 20 min to remove the inhibitor, prior to polymerization.

*Enzymes*: Heterologously produced and commercially available *Trametes versicolor* laccase (*Tve*Lac) was purchased from Sigma Aldrich (Merck, Darmstadt, Germany; ≥0.5 U/mg) and used as received. Laccase of the bracket fungus (polypore) *Pycnoporus cinnabarinus* (*Pci*Lac) was produced according to Eggert et al. [24]. *Aae*UPO and *Mro*UPO were produced and purified according to the protocols of Ullrich et al. [25] and Gröbe et al. [18], respectively. Recombinant UPO from *Agrocybe aegerita* (r*Aae*UPO, PaDa‐I variant) was produced and purified according to Molina‐Espeja et al. [26]. Specific activities of the enzyme preparations used in the present study are listed in Table 5.

**TABLE 5:** Specific activities of enzyme preparations (in units per mg protein—U/mg) used in this study.

Enzyme	Initial specific activity [U_VA_/mg]	Molecular weight [kDa] and corresponding reference^b^	
*Trametes versicolor* Laccase (*Tve*Lac)	≥0.5^a^	97	Han et al. [27]
*Pycnoporus cinnabarinus* Laccase (*Pci*Lac)	295	116	Eggert et al. [24]
*Agrocybe aegerita* UPO (*Aae*UPO)	82	46	Ullrich et al. [25]
recombinant *Agrocybe aegerita* UPO (r*Aae*UPO)	88	51	Molina‐Espeja et al. [26]
*Marasmius rotula* UPO (*Mro*UPO)	54	32	Gröbe et al. [18]

^a^ Information according to manufacturer.

^b^ Molecular weight according to mentioned references.

*Electron microscopy*: The TEM and cryo‐TEM imaging of Ag and hybrid particle systems was conducted with a Libra120 (Carl Zeiss Microscopy Deutschland, Germany), operating at 120 kV. For TEM, 2 µL of particle dispersion in water (1 mg/mL) were dropped on hydrophilized carbon film supported by a copper TEM grid and dried at ambient conditions. For cryo‐TEM, 2 µL of particle dispersion in water (1 mg/mL) were dropped on each side of hydrophilized QUANTIFOIL R3.5/1 TEM grid, blotted for 0.2 s and plunged in liquid ethane using Leica GP grid plunger (Leica Microsystems GmbH, Germany). Hydrophilization was done in air plasma at power of 70 W for 20 s (Femto Plasma Cleaner, Diener Electronic, Germany). To increase the contrast of the polymer brush, particles with PDMAEMA were stained (quaternized) with iodomethane. Therefore, 2 mL CH_3_I were added to 10 mg particles and stirred overnight. CH_3_I was removed by centrifugation and the particles redispersed in Millipore water.

The SEM investigation of Ag and composite particle systems was performed with a Volume Scope scanning electron microscope (Thermo Fisher Scientific GmbH, Germany), operating at 3 keV, ∼13 pA using T1 and secondary electrons detector. 2 µL of particle dispersion in water (1 mg/mL) were dropped on hydrophilized 5 mm × 5 mm silicon wafer (cleaned in a 1:1:1 mixture of H_2_O_2_, NH_4_OH, and H_2_O) and dried at room temperature. To enhance topography contrast, samples were coated with platinum (around 2 nm) using a Leica EM ACE 600 sputter coater (Leica Microsystems, Germany).

Moreover, SEM was conducted with an NEON40 (Carl Zeiss Microscopy Deutschland, Germany), operating at 2 keV, ∼200 pA using secondary electrons detector and back‐scattered electrons detector. For sample preparation, 2 µL of quaternized particle dispersion in water (1 mg/mL) was dropped on hydrophilized 5 mm × 5 mm silicon wafer and dried at ambient conditions and inspected without any coating. Hydrophilization of the wafer was done in air plasma at power of 70 W for 60 s (Femto, Diener Electronic, Germany).

*Electrokinetic measurements and dynamic light scattering (DLS)*: The pH‐dependent electrokinetic of the particles was investigated by Zetasizer Ultra Red from Malvern Instruments Ltd and an MPT‐3 autotitrator. A total of 24 mg particles were dispersed in 30 mL of 10 mM KCl solution. The pH of the prepared suspension was controlled by adding 0.1 M KOH or 0.1 M HCl using a titration system. Three measurements were recorded for each sample at each pH value. Same suspensions were used for the dynamic light scattering (DLS) measurements after full dispersion and measured three times for each sample.

*Synthesis of Ag particles*: According to previous literature reports [28], silver particles (170 nm) were synthesized in batches, using gold seeds. 5 mL of PVP (5 wt % in H_2_O) and 10 µL of HAuCl_4_ (0.25 M) were dissolved in 5 mL of H_2_O. After that, 0.6 mL of NaBH_4_ (0.1 M) was injected under vigorous stirring, giving rise to a yellowish solution of Au nanoparticles. The Au nanoparticles obtained were then aged for 6 h, allowing complete decomposition of NaBH_4_ before serving as the seeds in the subsequent seeded growth procedure. Two milliliters of PVP (5 wt % in H_2_O), 1 mL of acetonitrile, and 200 µL of ascorbic acid (0.1 M) were added in 2 mL of H_2_O, which was tempered to 25°C. Then, 150 µL of AgNO_3_ (0.1 M) was added, followed by quick injection of 0.1 µL of the seed solution. After 2 h, the particles were collected by centrifugation at 4000 rpm 20 min and washed three times with ethanol, redispersed in ethanol and stored in refrigerator.

*Synthesis of Ag‐Br‐Janus‐Particles (Ag@Br‐JP)*: 1 g of the Ag particles (about 170 nm in diameter) were dispersed in 100 mL chloroform and placed in a 250 mL round bottom flask with a reducer covered with aluminum foil with small holes. The particles were dispersed in an ultrasonic bath for 1 h. The ultrasonic bath was then heated to 50°C. Paraffin wax (10 g) were added and sonicated for an hour until the paraffin was completely dissolved. The chloroform was then carefully removed using a rotary evaporator (50°C: 450–200 mbar, 60°C: 200–80 mbar). The stirring blade and rod were inserted into the still liquid wax‐particle mixture and the flask was connected to a mechanical stirrer. The mixture was stirred at 85°C (water bath) and up to 800 rpm. Afterward, 100 mL of hot water (90°C) was added, and the stirring speed increased to 1100–1300 rpm. The emulsion was stirred at 85°C for 1 h. The water bath was then quickly removed, the stirrer switched off and the emulsion poured into two vessels with liquid nitrogen (2/3 filled). After the emulsion had been completely cooled down, it was transferred to filter papers with a plastic spatula, thawed and rinsed with DI water until the filtrate became clear. The filter papers were carefully dabbed dry from the outside with paper wipes and dried in vacuum at 25°C overnight.

After controlling in the SEM, the dried colloidosomes obtained were dispersed in 100 mL EtOH in a 250‐mL round‐bottomed flask. Afterward, 300 µL 11‐mercaptoundecyl 2‐bromo‐2‐methylpropanoate were added. After 2 days at <5°C and 400 rpm, the colloidosomes were poured into filter paper and rinsed with plenty of EtOH (approx. 1 L). The filter papers were carefully dabbed dry from the outside and dried in a vacuum at 25°C overnight.

The modified colloidosomes were placed in PTFE centrifuge tubes, filled with hexane, and dispersed in an ultrasonic bath at 60°C. The separation took place at 8000 rpm over 15 min. The waxy supernatant was collected in a beaker. The particles were washed six times with *n*‐hexane at 60°C, twice with DCM, and once with ethanol at RT and dispersed in 5 mL DMF for further synthesis.

*Synthesis of Ag‐PDMAEMA‐Janus‐Particles (Ag@PDMAEMA‐JP)*: 50 mg Ag@Br‐JP particles, 1 mL DMAEMA, 1.7 mL DMF, 6 µL CuBr_2_ solution (0.1 M in DMF), 1.3 mg TPMA, 0.03 µL EBIB were added to a sealed tube and purged with argon and sonicated (80 W) in an ice‐water bath for 10 min. Afterward, 20 µL Sn(II) 2‐ethylhexanoate dissolved in 0.2 mL DMF was injected. Polymerization of PDMAEMA on the Ag@Br‐JP surface was performed at 70°C in an oil bath for 60 min. After the reaction, the particles were collected by centrifugation at 4000 rpm for 20 min, washed twice in 30 mL DMF and three times in 30 mL ethanol, and dispersed in 10 mL ethanol.

*Synthesis of Ag particles with grafted PDMAEMA brush (Ag@PDMAEMA)*: First, ligand exchange was used to introduce Br‐initiator groups on silver particles by adding 300 µL 11‐mercaptoundecyl 2‐bromo‐2‐methylpropanoate to 200 mg Ag particles well dispersed in 100 mL ethanol. After stirring for 12 h, the Ag‐Br‐initiator was collected by centrifugation and washed three times with 30 mL ethanol. Finally, the Ag‐Br‐initiator was dispersed in 10 mL DMF and stored at 4°C.

Polymerization of PDMAEMA on the silver surface was performed in DMF at 70°C in an oil bath for 60 min. For this purpose, 50 mg Ag‐Br‐initiator particles, DMAEMA, 2 mL anhydrous DMF, 0.03 µL EBIB, 6 µL CuBr_2_ (0.1 M solution in DMF), 1.3 mg TPMA were added to a reaction‐tube with a stir bar, then the tube was sealed with a rubber septum and purged with argon and sonicated (80 W) in an ice‐water bath for 10 min. Three systems with DMAEMA monomer concentrations of 0.28, 0.99, and 1.7 M were synthesized for variation of the resulting chain length (Ag@PDMAEMA‐4 nm, ‐11 nm, and ‐23 nm). To this end, 0.1, 0.4, or 0.8 mL of the monomer were added to the reaction mixture. Afterward, 20 µL Sn(II) 2‐ethylhexanoate dissolved in 0.2 mL DMF were injected. After the reaction, the particles were collected by centrifugation at 4000 rpm for 20 min, washed two times in 30 mL DMF and three times in 30 mL ethanol and dispersed in 10 mL ethanol.

*Immobilization of laccase in the polymer brush of the synthesised particles*: First, 10 mg PDMAEMA grafted particles were dispersed in 3 mL buffer (10 mM sodium acetate, Sodium acetate) solution by sonication until no particles were visible at the bottom of the centrifuge tube (it took about 1 min). After washing with 3 mL buffer solution (three times), the particles were redispersed in 0.5 mL buffer solution and mixed with 0.5 mL dissolved laccase (∼50 U in 10 mM sodium acetate buffer) The immobilization was carried out for one hour under gentle shaking at room temperature. Afterward the particles were washed with buffer until no activity was found in the supernatant.

Determination of enzyme activity was performed with a plate reader (Tristar 5 Multimode, Berthold Technologies). Laccase oxidizes 2,2′‐azino‐*bis*(3‐ethylbenzothiazoline‐6‐sulfonic acid) (ABTS) to a blue‐green cation‐radical (ABTS**^+^** *). To start the reaction, 1 µL of the enzyme solution was added to 974 µL sodium acetate buffer (10 mM, pH 4.0) in the reaction well. During measurement, 25 µL of 2 mM ABTS solution were added by the injection unit of the plate reader. Colorimetric changes were measured by following the absorption at 420 nm. The activity can be calculated from the measurement of the change in absorbance (Δ*E*) within a particular time interval (Δ*t*) as already reported [10b,c].

*Immobilization of rAaeUPO and MroUPO in the polymer brush of the synthesised particles at different* pH *values*: Each 20‐mg batch (dispersed in EtOH) was treated in an ultrasonic bath for 6 min. The 20 mg inserts were divided into 3 mg aliquots. The aliquots were centrifuged at 6000 rpm for 5 min and then dispersed in 50 mM sodium acetate buffer pH 4.0 or 6.0. This procedure was repeated three times. Each aliquot was then centrifuged and dispersed in 500 µL of an enzyme solution containing 600 U/mL UPO (300 U in total) at pH 4.0 or 6.0. The samples were incubated for 2 h at 10°C while shaking at 300 rpm to prevent settling of the particles. After 2 h, the samples were centrifuged at 6000 rpm, the supernatant was removed and stored for later measurement of the enzyme activity. The aliquots were washed until no more activity was present in the supernatant (usually, three washings steps were sufficient). The washed particles were used for further analysis and tests. To test the behavior of the core‐shell particles at different pH values, the particles were dispersed in the buffers at pH 4.0 and 6.0.

*“Cleaning” and reuse of particles*: Particles successfully loaded with enzyme (approx. 10 mg) were centrifuged at 6000 rpm for 5 min, and the supernatant was removed. The particles were resuspended in 1 mL of 50 mM sodium acetate with 1 M sodium chloride and incubated in an ultrasonic bath for 20 min (with temperature control at 20°C to avoid overheating). The salt was washed off, and the particles were resuspended in 50 mM sodium acetate. After resuspension, no catalytically active UPO was found on the particles. The particles were divided into three aliquots of about 2 mg each. The immobilization of *Mro*UPO on the cleaned particles was then carried out as described above with some modifications. The initial amount of enzyme was varied in three steps, using 50, 100, or 150 U_VA_/mL (veratryl alcohol oxidizing units, see below), to assess whether the initial enzyme activity during immobilization affects the final product.

*Determination of protein loads and enzymatic activities on immobilized particles*: The protein load of particles with successfully immobilized enzymes, that is, Ag@PDMAEMA‐JP (with sufficient activity and good dispersion), was determined using a standard protein assay according to Bradford [29]. An untreated preparation of the Ag@PDMAEMA‐JP was used as the blank. The preparation contained about 10 mg particles/mL (= 1 mg particles/100 µL) and gave a Bradford value of 0.35 mg/mL (including dilution). UPO activities toward veratryl alcohol (U_VA_) were measured photometrically at 310 nm on a Varian Cary 50 spectrophotometer (Agilent, USA) using the standard UPO assay containing 5 mM veratryl alcohol, 50 mM potassium phosphate buffer (KP_i_) at pH 6.0 and initializing the reaction with mM H_2_O_2_ (Ullrich et al.) [25].

*Comparing* pH*‐profiles of free and immobilized UPOs*: The pH‐dependent oxygenation of veratryl alcohol into veratraldehyde was determined photometrically using the UPO standard assay at varying pH between 4.0 and 8.0 in steps of 0.5 [25].

To determine whether the shift in the pH optimum is due to the detachment of the enzymes from the particles as an effect of the pH, a further experiment was carried out. The setup was as follows: 50 mM KP_i_, 5 mM veratryl alcohol, 25 µL *Mro*UPO/Ag@PDMAEMA‐JP solution were prepared in a total of 1 mL. In the supernatants (950 µL), enzyme activity was measured at 310 nm after centrifugation at 6000 rpm for 12 min and addition of 10 µL (100 mM) H_2_O_2_ according to the standard assay.

## Author Contributions

The manuscript was written through contributions of all authors. All authors have given approval to the final version of the manuscript. AK and HC contributed equally.

## Conflicts of Interest

The authors declare no conflicts of interest.

## Data Availability

The data that support the findings of this study are available on request from the corresponding author. The data are not publicly available due to privacy or ethical restrictions.
